# Salt tolerance threshold and physiological responses in Bach Thao goats drinking diluted seawater under tropical conditions

**DOI:** 10.14202/vetworld.2023.1714-1720

**Published:** 2023-08-24

**Authors:** Thiet Nguyen, Khang Van Truong, Ngu Trong Nguyen, Sumpun Thammacharoen

**Affiliations:** 1 Department of Agricultural Technology, College of Rural Development, Can Tho University, 3/2 Street, Can Tho city 94000, Vietnam; 2 Department of Animal Science, College of Agriculture, Can Tho University, 3/2 Street, Can Tho city 94000, Vietnam; 3 Department of Physiology, Faculty of Veterinary Science, Chulalongkorn University, HenriDunang Street, Bangkok 10330, Thailand

**Keywords:** body weight, climate change, freshwater, salt tolerance

## Abstract

**Background and Aim:**

Climate change challenges with incremental sea level cause saltwater intrusion, which has affected the quality of freshwater and groundwater in coastal provinces, particularly the Mekong River Delta provinces of Vietnam. Interestingly, Bach Thao goats are predominant in this area and well adapted to saline water under tropical conditions. Therefore, this study investigated the salt tolerance threshold of Bach Thao goats drinking diluted seawater (DSW).

**Materials and Methods:**

The experiment was performed using seven Bach Thao male goats (20.60 ± 1.12 kg) and divided into two phases. In Phase 1 (control, C), all goats were provided fresh water (FW) for 7 days from two identical buckets, and daily water intake (WI) was recorded from both buckets. In Phase 2 (preference test, PT), each goat was provided FW from the first bucket and DSW from the second bucket or vice versa. The concentration of DSW for the preference test was 0.5%–2.0%.

**Results:**

Body weight and dry matter intake showed no differences according to DSW consumption; however, WI was significantly lower during Phase 2 (p < 0.05), which was due to the lower WI with 1.5% and 2% of DSW (p < 0.05). Goats showed a similar preference for fluid intake between FW and DSW at 0.0%–1.0% levels and began avoiding DSW at 1.5% and rejected at 2.0% of DSW. Goats consuming 1.5% of DSW showed increased respiration rate from 13:00 to 19:00 h and rectal temperature at 13:00 h (p < 0.05).

**Conclusion:**

Goats can tolerate up to 1.0% of DSW. Shifting to FW activated aversive drinking to 1.5% and 2.0% of DSW. This behavioral response was prominent at 0.5% DSW. Moreover, goats that drank 1.5% of DSW had decreased thermoregulation.

## Introduction

Agriculture in Vietnam has vigorously developed for several years, including pig, cattle, and poultry production. However, goat farms have become increasingly popular in recent years. They have existed for a long time, primarily by extensive farming systems [1]. Nevertheless, climate challenges with incremental sea level cause saltwater intrusion, which has impacted the quality of fresh water (FW) and groundwater in coastal provinces, particularly the Mekong River Delta (MRD) provinces of Vietnam. Based on estimates, if the sea level increases 1 m, approximately 39% of the area of MRD would be at risk of salinity intrusion. It has been reported that in the coastal provinces in MRD, the salinity levels of surface water in some locations were 0.6%–1.5% [2]. Consequently, drinking water for animals is contaminated with salt, affecting animal production.

The previous studies by Silanikove [3] have indicated that goats can adapt to salt-contaminated water; for instance, in arid conditions, small ruminants can drink water with high salt concentration and even survive on seawater [4]. McGregor [5] suggested that goats tolerate higher saline water when green herbage and shade are provided. Furthermore, other factors influence salt tolerance in animals, such as age [6, 7], physiological adaptation and thermal stress [8], or breed [9]. Moreover, sheep and goat consuming saline water control the salt load by excreting more urine volume, whereas camels decrease the salt stress by drinking less saline water [10]. Experiments on lactating crossbred Saanen goats suggested that goats that consumed saline water had increased water intake (WI) at 0.0%–1.0% saline water and decreased WI at 1.5% saline water [11]. Some studies have used a two-choice preference test that describes taste responses or thresholds to saline drinking water in goats [6], sheep [12, 13], and cattle [14]. However, there is limited information on the effect of diluted seawater (DSW) on salt tolerance threshold and physiological responses in goats under tropical conditions.

Therefore, this study was conducted to investigate the effect of DSW on salt tolerance threshold and physiological responses in goats under tropical conditions. We hypothesized that Bach Thao goats can tolerate up to 1.5% of DSW by changing their preference for FW.

## Materials and Methods

### Ethical approval

The study was approved by the Scientific Committee of Can Tho University (#3559).

### Study period and location

The study was conducted from October to December 2021 in Experimental Farm, College of Rural Development, Can Tho university.

### Experimental design and animal care

Seven Bach Thao male goats aged approximately 7 months and weighing 20.60 ± 1.12 kg were used in this study. The number of animals and experimental design was established according to the previous studies by Runa *et al*. [6], Goatcher and Church [12], and Goatcher and Church [13]. One week before the start of the experiment, the goats were maintained in cages (each measuring 2 × 2 m with a plastic floor) for adaptation and divided into two phases. In Phase 1 (control, C), all goats were provided FW for 7 days from two identical buckets, and daily WI was recorded from the two buckets. We provided 5 kg of water in each bucket before morning feeding and refilled it before afternoon feeding. In Phase 2 (preference test, PT), each goat was provided FW from the first bucket and DSW from the second bucket or vice versa. The location of the bucket was decided according to recommendations from the previous studies by Runa *et al*. [6], Goatcher and Church [12], and Goatcher and Church [13], and it was changed daily at 07:00, 13:00, and 18:00 h to avoid errors due to the goat’s habit of recognizing the location of the bucket that contains FW or DSW. Each experimental concentration was evaluated for 48 h (2 consecutive days). This procedure allows us to determine the threshold at which goats partially or completely accept or reject saline drinking water. The preference test (Phase 2) began with 0.5% (day 8–9), 1.0% (day 10–11), 1.5% (day 12–13), and 2.0% total dissolved solids (TDS) of DSW levels (day 14–15). The threshold at which goats refused to drink salt water was when they drank <20% of the total fluid intake from both buckets provided to each goat [13]. The DSW used in this experiment was prepared by dilution between concentrated seawater (9%) from the aquaculture farm and FW (Table-1). Briefly, concentrated seawater (9%) was mixed with FW to achieve water with a salt concentration of 0.5%, 1.0%, 1.5%, or 2% according to the formula C1V1 = C2V2 (where C1 is the concentration of the starting solution; V1 is the volume of the starting solution; C2 is the concentration of the final solution; and V2 is the volume of the final solution). The salinity level was directly checked using a refractometer (Master S28M, Atago, Japan).

**Table-1:** Chemical composition of fresh water and concentrated seawater from present experiment.

Items	Fresh water	Concentrated seawater
EC (mS/cm)	0.28	214
TDS (mg/L)	127	97,000
CL^-^ (mg/L)	28	63,340
K^+^ (mg/L)	4.35	1,110
Na^+^ (mg/L)	16.6	31,972
Ca^2+^ (mg/L)	15.5	575
Mg^2+^ (mg/L)	9.91	4,109

EC=Electrical conductivity, TDS: Total dissolved solids

The chemical compositions of FW and concentrated seawater are shown in Table-1. The goats consumed 0.4 kg concentrate and had free access to natural grass according to the farm’s protocol. The chemical composition of the experimental diets is presented in Table-2. Humidity and air temperature (TR) were recorded on days 5 and 7 at Phase 1 and days 11 and 13 at Phase 2, and then, the temperature and humidity index (THI) was calculated.

**Table-2:** Chemical composition of experimental diets (DM basis).

Items	Concentrate (%)	Natural grass (%)
DM	87.13	20.52
CP	18.85	7.61
ADF	20.02	44.32
NDF	38.91	62.50
Ash	7.97	9.67

DM=Dry matter, CP=Crude protein, ADF=Acid detergent fiber, NDF=Neutral detergent fiber

### Data collection and analysis

Feed intake was measured daily from days 1–7 for Phase 1 and days 8–15 for Phase 2. Feed was provided and refusal samples were collected daily and stored in the freezer for later analysis. At the end of the experiment, all samples were thawed, mixed, and divided into two parts; the first part was dried at 105°C in an oven to determine dry matter, and the second part was dried at 60°C for chemical composition analysis. Proximate analysis of feed was performed as described previously by Association of Official Analytical Chemists [15], and neutral detergent fiber and acid detergent fiber were evaluated using the procedure described by Van Soest *et al*. [16].

WI was measured daily for Phase 1 and Phase 2. All the goats were weighed at morning feeding on days 0, 7, and 15 of the experiment. The analysis of water samples and TDS was performed as described in our previous experiment by Nguyen *et al*. [17]. Briefly, the mineral composition of water samples was determined by atomic absorption spectrophotometry (Thermo iCE3000 series, Thermo Fisher Scientific, China). Chloride content was determined by the titration of 0.02 N AgNO_3_. EC was measured using an EC meter (Schott Instruments D-55122, Mainz, Germany) and used to calculate TDS = EC (mS/cm) × 0.454. The percentage of fluid intake was calculated using the following formula:

Percentage of fluid intake (%) = (amount of DSW the goats drank, kg) × 100/total amount of fluid intake (kg) in the two buckets.

Rectal temperature (RT) and respiration rate (RR) were measured at 07:00, 09:00, 11:00, 13:00, 15:00, 17:00, and 19:00 h on day 7 at Phase 1 and on days 9, 11, 13, and 15 at Phase 2. RT was measured using a digital clinical thermometer (C202, Terumo, Tokyo, Japan), and RR was measured by counting flank movements within 1 min.

### Statistical analysis

All data are expressed as mean ± standard error of mean. The average values of body weight (BW), dry matter intake (DMI), WI, RT, and RR were calculated for Phase 1 and Phase 2 and then analyzed by paired t-test between both phases. The average WI of the preference test was determined from 2 consecutive days in the same fluid concentration (FW or DSW) and, then, analyzed by paired t-test between FW and DSW. The values of daily WI, RT, and RR across the treatment phases were analyzed by one-way analysis of variance. The significance level was set at p < 0.05, and a tendency was established at 0.05 < p < 0.10.

## Results

### Environmental conditions during the experiment

Table-3 shows the environmental conditions during Phase 1 and Phase 2 of the present study. The average ambient temperature (Ta) and THI between Phase 1 and Phase 2 were low at 07:00 and 19:00 h (27.5°C and 28.0°C for Ta and 78.77 and 78.59 for THI, respectively) and high at 13:00 and 15:00 h (31.5°C and 32.2°C for Ta and 82.12 and 83.43 for THI, respectively). However, the relative humidity percentages were high at 07:00 h (78.25%) and low at 13:00 h (60.0%) in this study.

**Table-3:** Environmental conditions during Phase 1 and 2 from present experiment.

Time	Phase 1			Phase 2		
	
	T_a_ (°C)	Humidity (%)	THI	T_a_ (^0^C)	Humidity (%)	THI
07:00	27.50 ± 0.50	79.00 ± 1.00	78.87 ± 0.67	27.50 ± 0.41	77.50 ± 3.67	78.67 ± 0.19
09:00	29.00 ± 1.00	68.00 ± 3.00	79.70 ± 1.06	29.00 ± 0.82	68.50 ± 0.41	79.79 ± 1.16
11:00	30.00 ± 1.00	60.50 ± 2.50	80.07 ± 1.03	30.00 ± 0.82	65.00 ± 0.82	80.77 ± 1.31
13:00	31.50 ± 0.50	56.50 ± 1.50	81.54 ± 0.44	31.50 ± 0.41	63.50 ± 1.22	82.71 ± 0.79
15:00	32.25 ± 0.25	59.50 ± 0.50	83.08 ± 0.26	32.20 ± 0.16	64.00 ± 0.82	83.79 ± 0.38
17:00	29.50 ± 0.50	64.00 ± 1.00	79.90 ± 0.87	28.75 ± 0.20	73.00 ± 4.08	80.03 ± 0.25
19:00	28.50 ± 0.50	65.00 ± 2.00	78.57 ± 0.46	27.50 ± 0.41	77.00 ± 1.63	78.62 ± 0.44

T_a_=Ambient temperature, THI=Temperature and humidity index

### Body weight, DMI, and WI

**Figure-1:**
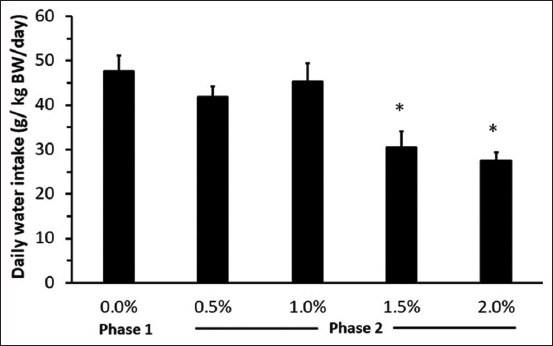
Total daily water intake from Phase 1 (fresh water FW/FW) and Phase 2 (FW/DSW). *Significant difference between each DSW concentration from Phase 2 and Phase 1 (p < 0.05). FW=Fresh water, DSW=Diluted seawater.

Body weight and DMI remained constant between Phase 1 and Phase 2 (p > 0.05; Table-4). However, the total WI (g/kg BW/day) in Phase 2 was significantly lower than that in Phase 1 (p < 0.05; Table-4). The total WI from 1.5% and 2.0% of DSW was significantly lower than that from 0.5% and 1.0% of DSW (Figure-1; p < 0.05).

**Table-4:** Body weight, dry matter intake, and water intake during the control (Phase 1) and treatment phases (Phase 2).

Items	Experimental phase		p-value

	Phase 1 (C)	Phase 2 (PT)	
BW (kg/head)	20.60 ± 1.12	21.86 ± 1.20	0.46
DMI (kg/head/day)	0.65 ± 0.01	0.64 ± 0.01	0.66
DMI (g/kg BW/day)	32.11 ± 1.79	30.09 ± 1.81	0.44
WI (kg/head/day)	0.98 ± 0.09	0.78 ± 0.03	0.053
WI (g/kg BW/day)	47.72 ± 3.40	36.28 ± 1.91	0.013

BW=Body weight, DMI=Dry matter intake, WI=Water intake

**Figure-2:**
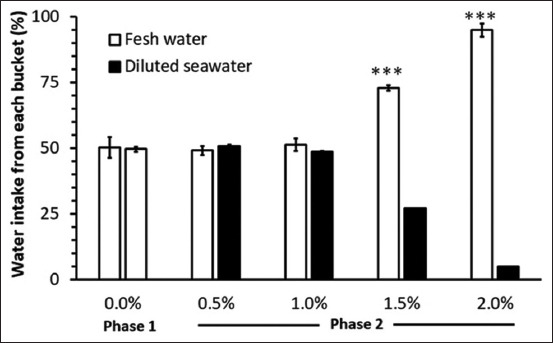
The percentage of fluid intake from two buckets of preference test from Phase 1 (FW/FW) and Phase 2 (FW/DSW). *Significant difference in water intake between FW and DSW from each preference test day (p < 0.05). FW=Fresh water, DSW=Diluted seawater.

Throughout the treatment phase, as shown in Figure-2, all goats significantly preferred FW over DSW. There were no differences in preference between FW and DSW at 0.5% and 1.0% of concentrations. However, at 1.5% of DSW, the goats preferred FW (72.88%). They rejected water at 2.0% of DSW and preferred drinking FW (94.90%).

### Rectal temperature and RR

Temperature showed no difference between Phase 1 and Phase 2 throughout the daytime, except at 13:00 h where the RT in Phase 2 was higher than in Phase 1 (p < 0.05; Table-5). Similarly, RR remained unaffected between the control and treatment phases from 07:00 to 11:00 h. However, RR in Phase 2 was greater than that in Phase 1 from 13:00 to 19:00 h (p < 0.05; Table-5). Across the treatment phases, RT remained unaffected by DSW from 07:00 to 11:00 h and 17:00 to 19:00 h (Table-6). However, the RT of goats drinking 1.5% DSW was higher than that of goats drinking either 0.0% or 0.5% of DSW from 13:00 to 15:00 h (p < 0.05; Table-6). Respiration rate was similar among treatments from 07:00 to 09:00 h; at 15:00 h, the animals consuming 1.5% DSW had higher RR than animals consuming 0.0% and 0.5% of DSW from 11:00 to 13:00 h and from 17:00 to 19:00 h (p < 0.05; Table-6).

**Table-5:** Rectal temperature and respiration rate during the control (Phase 1) and sensitivity test (Phase 2).

Items	Time (h)	Experimental phase		p-value

		Phase 1 (C)	Phase 2 (PT)	
Rectal temperature (°C)	07:00	38.70 ± 0.06	38.59 ± 0.07	0.26
	09:00	38.97 ± 0.06	39.00 ± 0.05	0.71
	11:00	39.04 ± 0.10	39.01 ± 0.04	0.75
	13:00	38.76 ± 0.14	39.14 ± 0.04	0.02
	15:00	38.81 ± 0.16	39.01 ± 0.06	0.26
	17:00	38.89 ± 0.11	39.12 ± 0.06	0.08
	19:00	39.10 ± 0.08	39.14 ± 0.04	0.68
Respiration rate (breath/min)	07:00	46.43 ± 1.60	45.00 ± 1.04	0.47
	09:00	59.00 ± 3.43	55.39 ± 1.31	0.35
	11:00	68.00 ± 2.25	69.93 ± 0.88	0.44
	13:00	70.43 ± 1.00	79.11 ± 2.72	0.01
	15:00	63.57 ± 2.31	69.00 ± 1.43	0.07
	17:00	51.43 ± 1.53	64.61 ± 0.91	0.001
	19:00	49.71 ± 2.45	61.82 ± 1.12	0.001

**Table-6:** Rectal temperature and respiration rate across the phases (Phase 1 and 2).

Time (h)	0.0%	0.5%	1.0%	1.5%	2.0%	SE	p-value
Rectal temperature (°C)							
07:00	38.70	38.24	38.86	38.53	38.74	0.15	0.08
09:00	38.97	38.86	39.06	38.93	39.16	0.09	0.20
11:00	39.04	38.97	39.06	38.89	39.11	0.10	0.56
13:00	38.76^b^	39.16^ab^	39.03^ab^	39.24^a^	39.11^ab^	0.11	0.04
15:00	38.81^ab^	38.73^b^	39.01^ab^	39.21^a^	39.09^ab^	0.12	0.04
17:00	38.89	39.04	38.97	39.30	39.16	0.10	0.07
19:00	39.10	39.17	39.00	39.21	39.16	0.07	0.32
Respiration rate (breath/min)							
07:00	46.43	43.29	46.71	43.43	46.57	1.74	0.41
09:00	59.00	49.86	61.57	55.86	54.29	4.16	0.17
11:00	68.00^ab^	62.29^b^	70.14^ab^	82.86^a^	64.43^ab^	4.16	0.01
13:00	70.43^b^	73.71^ab^	78.86^ab^	86.14^a^	77.71^ab^	3.44	0.04
15:00	63.57	68.57	68.71	72.57	66.14	3.58	0.49
17:00	51.43^b^	60.43^ab^	65.86^a^	69.43^a^	62.71^a^	2.40	0.001
19:00	49.71^b^	51.71^b^	63.57^a^	67.86^a^	64.14^a^	2.58	0.001

0.0%: Fresh water; diluted seawater concentration at 0.5%; 1.0%; 1.5%; and 2.0%. ^a,b^Means within the same row with different superscripts differ significantly at p *<* 0.05. SE=Standard error

## Discussion

There was no difference in the preference of WI between FW and DSW at a concentration of up to 1.0%. The preference for DSW at concentrations of 1.5% and 2% was lower than that for FW. Furthermore, consumption of 1.5% and 2% of DSW affected total WI, and Bach Thao goats adapted to these concentrations. When the total WI was decreased under the experimental conditions, Bach Thao goats adapted their thermoregulation by increasing their TR and RR.

### Dry matter intake and WI

Dry matter intake showed no difference between Phase 1 and Phase 2, although Bach Thao goats from Phase 2 consumed a large amount of minerals (Na, K, Cl, Ca, and Mg) from DSW compared with goats from Phase 1 (FW). In principle, the animals that drank saline water would have increased Na^+^ and K^+^ intakes and may increase ruminal osmolality. Baile and Mayer [18] found that infusion of Na acetate into the reticulorumen may increase osmolality, reducing feed intake. In contrast, injecting the same amount of Na acetate into the jugular vein did not affect DMI. Furthermore, Potter *et al*. [19] reported that sheep drinking saline water had higher WI and osmotic pressure in the rumen. Nevertheless, the results of the present study demonstrated that Bach Thao goats adapted to saline water by either decreasing their WI from high saline levels (1.5% and 2%) to avoid salt stress or increasing their WI from low (0.5%) to moderate (1.0%) saline water (Figures-1 and 2) or excreting higher urinary electrolytes as mentioned previously by Nguyen *et al*. [17]. The result of DMI was similar to that of previous studies on Boer crossbred goats [17], sheep [20], and deer [21] and. In contrast, a lower DMI was found in 2% of saline water [22]. Some studies showed that DMI increased gradually when animals drank low saline water such as that in rusa deer [23] and Boer goats [24]. The different responses in DMI, when animals drank saline water, may differ according to species or saline levels. Body weight remained unaffected by salinity levels in drinking water for a short period (15 days); this result was similar to the previous studies by Peirce [20], and Zoidis and Hadjigeorgiou [22] when the animals drank 1% of saline water, whereas the BW decreased when animals consumed 2% of NaCl. Runa *et al*. [6] demonstrated that the BW of crossbred Boer goats showed no changes after drinking saline water. During the experimental period, Bach Thao goats could maintain both BW and DMI. These data suggest that the goats could tolerate the sequential increase in DSW concentration and any effect caused by DSW is not indirect by changing DMI.

Bach Thao goats showed decreased WI from 1.5% of DSW (approximately 25% from the control) or rejected drinking 2% of DSW (approximately 45% from the control) and similar WI at 0.5% and 1.0% of DSW levels (Figure-2). Therefore, the total WI in Phase 2 was lower than that in Phase 1 in this experiment. Total WI should not differ among treatments in our experiment (Figure-1) because, during the preference test of Phase 2, all goats could access FW as a choice. If the effect of DSW in Phase 2 is true, the result suggested that a high DSW concentration could change the total WI from a behavioral or physiological viewpoint. Although we did not determine behaviors such as lying, standing, and time for eating or drinking, we observed that Bach Thao goats drinking 1.5% or 2.0% of DSW spent more time for panting (Table-5), lying, or standing and not for drinking. This phenomenon indicated that animals feel uncomfortable and alter their behaviors to adapt to 1.5% or 2.0% of DSW. This finding was in contrast to that of a previous study on llamas [7], probably due to different species (llamas and local goats) and environmental conditions (temperate and tropical conditions). The impact of 1.5% and 2.0% of DSW on both total WI and preference tests in the present study suggests that Bach Thao goats could rapidly detect saline water contamination by an aversive response. Moreover, the high DSW concentrations (1.5% and 2.0%) apparently have a behavioral effect on total WI. The latter phenomenon needs further investigation in the context of the neurobehavioral mechanism.

In the preference test, Bach Thao goats drank FW, which may balance the mineral intakes (Na, K) from DSW and avoid increasing body water. The animal’s ability can tolerate various degrees of salt loads in drinking water, which may relate to kidney function [25] by increasing the excretion of renal electrolytes [17], and decreasing or rejecting high saline water. Similarly, a previous study by Nguyen *et al*. [11] suggested that lactating crossbred Saanen goats consumed more saline water at concentrations of 0.0%–1.0% but decreased their WI at 1.5% of saline water. Accordingly, Bach Thao goats can balance the excess electrolyte intake by preferring either FW or DSW and maintaining it in an acceptable range. Nevertheless, the results showed that fresh WI was greater than high DSW intake due to the ability of goats to distinguish between FW and different levels of DSW. Goats prefer 0.5% and 1.0% of DSW, but they drank <20% of the total daily intake at 2% of DSW, indicating a rejection threshold [12, 13]. Goats could accept 1.5% of NaCl in drinking water [5], but Boer goats rejected water containing 1.25%–1.5% of NaCl [6]. Enke *et al*. [7] found that llamas had a weak preference for saline water containing 0.5%–0.75% of NaCl and rejected saline water containing 1.25% of NaCl. Abou Hussien *et al*. [10] reported that camels were more tolerant to saline water than goats and sheep because they exhibited low WI and decreased urinary volume. In deer, the salt tolerance was 0.8%–1.2% [23]. Salt tolerance showed large differences among species, with the following order: Camels > goat = sheep > llamas = deer. In the present study, Bach Thao goats could tolerate up to 1.0% of DSW.

### Rectal temperature and RR

At high ambient temperature under tropical conditions, RT increases according to daytime, as reported previously by Saipin *et al*. [26]. In the present study, the RT of Bach Thao goats in Phase 2 was higher than in Phase 1 at 13:00 h and tended to be higher at 17:00 h. This result indicated that the RT of goats that consumed FW or 0.5% DSW remained unaffected, whereas the RT of goats that drank 1.5% DSW increased during the hottest time of the day (13:00–15:00 h, THI 82–83). Interestingly, goats that drank 2% of DSW had similar RT as that of goats that drank FW, which may be because the former group of goats adjusted their drinking behavior by rejecting WI (<20%) to decrease the salt stress. Therefore, the total electrolyte intake from 2% DSW was apparently <1.5% in this experiment. A higher RT from a saline diet may be due to greater heat production for urinary mineral excretion, as suggested previously by Arieli *et al*. [24]. Similar energy expenditure with 1.5% of DSW may be found in this experiment. Similar results were reported in the previous studies by Eltayeb [27] and Mdletshe *et al*. [28], where RT increased due to saline water consumption. However, Mdletshe *et al*. [28] found that 0.55%–1.1% of saline water did not affect the RT of goats at 08:00 h, and the present study also showed similar findings from 07:00 to 11:00 h but differences in RT at 13:00 and 15:00 h. The changes in RR were affected by either environmental conditions during the daytime or DSW levels in this experiment. Respiration rate was higher in Phase 2 than in Phase 1 in the afternoon (from 13:00 to 19:00), but it remained unaffected by saline water during the cooling time of day (morning). Runa *et al*. [6] reported that the RR of Boer goats in the sensitivity test at 11:00 and 15:00 h was unaffected by saline water when exposed to temperate conditions. Similarly, we also found that the RR of Bach Thao goats remained unaffected by DSW in the morning. In contrast, Mdletshe *et al*. [28] reported that 1.1% of saline water increased the RR of Nguni goats at 08:00 h. The higher TR and RR observed in the present study indicated that Bach Thao goats that drank 1.5% of DSW had decreased thermoregulation efficiency under the high ambient temperature of tropical conditions and were less tolerant than goats that drank 0.5% and 1.0% of DSW.

## Conclusion

Bach Thao goats can tolerate up to 1.0% of DSW. High concentrations of DSW (1.5%–2%) negatively influenced the total WI and subsequently decreased thermoregulation. With the sequential increase in DSW concentrations, Bach Thao goats could differentiate the concentration of saline contamination at 0.5%, for example, 1.0% from 1.5%, by an aversive response.

## Authors’ Contributions

TN, NTN, KVT, and ST: Contributed to the conception and design of the study. TN, KVT, and NTN: Contributed reagents/materials/analysis tools. TN and KVT: Performed animal experiments. NT and ST: Statistical analysis and wrote and revised the manuscript. All authors have read, reviewed, and approved the final manuscript
